# Pattern of antigen expression in metastases after radioimmunotherapy of a syngeneic rat colon carcinoma utilizing the BR96 antibody

**DOI:** 10.1186/2162-3619-1-34

**Published:** 2012-11-13

**Authors:** Erika Elgström, Sophie E Eriksson, Tomas G Ohlsson, Jan Tennvall, Rune Nilsson

**Affiliations:** 1 Department of Oncology, Lund University, Lund, Sweden; 2 Department of Medical Radiation Physics, Lund University, Lund, Sweden; 3 Department of Oncology, Skåne University Hospital, Lund, Sweden

**Keywords:** Radioimmunotherapy, Rat colon carcinoma, ^177^Lu, Antigen expression, Metastasis

## Abstract

**Background:**

Repeated administration of antibody-based therapies such as radioimmunotherapy depends on preserved antigen expression in tumor lesions. The purpose of this study was to evaluate whether the antigen expression in metastases observed after radioimmunotherapy differs from that of untreated primary tumors.

**Findings:**

30 of the 35 Brown Norway rats with syngeneic colon carcinoma treated with 400 MBq/kg ^177^Lu-DOTA-BR96 exhibited consistent complete response of the primary tumor. 13 animals developed metastases that were detected after treatment. The antigen expression was reduced in 17 of 23 metastases detected after radioimmunotherapy compared with untreated tumors. No tumors completely lacked positively stained tumor cells.

**Conclusions:**

Although it was not possible to demonstrate that the antigen reduction is triggered by the radioimmunotherapy this result stress the importance of considering the risk of reduced antigen expression in metastases after radioimmunotherapy prior to further targeted therapies.

## Background

Treatment of primary tumors is often successful compared to that of metastatic disease, which is responsible for 90% of cancer mortality [1]. Radioimmunotherapy, the use of radiolabeled antibodies to localize radiation to the tumor, is considered a suitable treatment modality for smaller metastases [2]. Repeated administrations of radioimmunoconjugate may be required and/or beneficial in order to reduce toxicity and improve the outcome [3].

The aim of the present study was to compare the antigen expression and distribution in metastases, detected after consistent complete response (CR) of the primary tumor following radioimmunotherapy with ^177^Lu-DOTA-BR96, to that of untreated primary tumors in a syngeneic immunocompetent rat colon carcinoma model.

## Findings

### Radioimmunoconjugate and tumor model

BR96 is a monoclonal antibody which binds to the Lewis Y antigen (Le^y^). Conjugation with the DOTA-chelate (S-2-(4-isothiocyanatobenzyl)-1,4,7,10-tetraazacyclododecane tetraacetic acid; Macrocyclics, Dallas, TX) and subsequent radiolabeling with ^177^LuCl_3_ solution (MDS Nordion, Vancouver, Canada) was performed according to Eriksson *et al*. [4]. The radiochemical purity of ^177^Lu-DOTA-BR96 was analyzed using ITLC and found to be >97.5%. Less than 1.3% labeled aggregated fractions was detected with HPLC.

Brown Norway (BN) rats were used (Harlan, Horst, Netherlands). Animals of this rat strain are immunocompetent and express the BR96 binding antigen (Lewis Y) in some normal tissues, mainly in the epithelium of the gastrointestinal tract [5], and therefore mimic the clinical situation.

The animals were inoculated under anesthesia (Isoflurane, Abbott Scandinavia AB, Solna, Sweden) with 3 x 10^5^ BN7005-H1D2 cells (a cell line established from a 1,2-dimethylhydrazine-induced colon carcinoma in a BN rat), between the peritoneum and the abdominal wall. The resulting local tumors were designated primary tumors. Tumor volumes were calculated as tumor length x tumor width^2^ x 0.4. All experiments were conducted in compliance with Swedish legislation on animal rights and protection, and were approved by the Regional Animal Ethics Committee. The animals were housed under standard conditions and fed with standard pellets and fresh water, *ad libitum*.

### Radioimmunotherapy with ^177^Lu-DOTA-BR96

Thirty-five male rats were treated with 400 MBq/kg ^177^Lu-DOTA-BR96 (150 μg DOTA-BR96 in 0.4 ml saline with 1% human serum albumin) by intravenous injection 13-14 days after cell inoculation Group A). 11 tumor-bearing rats were not treated (Group B). The treated animals were monitored for up to 120 days after injection of ^177^Lu-DOTA-BR96. The control animals (Group B) were euthanized and dissected on the day of administration of radioimmunotherapy to Group A (day 0). The median primary tumor volume on the day of treatment was 490 mm^3^ (range 115-1490 mm^3^) and the mean body weight was 253 g (SD 23 g).

Twice a week, body weight of the animals in Group A was recorded, and the diameters of the primary tumors were measured with a digital caliper under anesthesia Consistent CR was defined as a non-palpable tumor during the rest of the observation period. Rats were sacrificed if the dimensions of the tumor exceeded 25 x 25 mm, or at the end of the study (120 days). If body weight decreased by more than 20% compared to normal weight progression, or if the general health of the animal was affected during the study, metastatic disease was suspected and the rat was sacrificed and dissected to confirm metastatic growth. At the time of sacrifice, all rats were dissected systematically by the same person, and the location and number of metastatic sites was registered. Tumor findings were fixed in 4% paraformaldehyde and embedded in paraffin.

**Figure 1:**
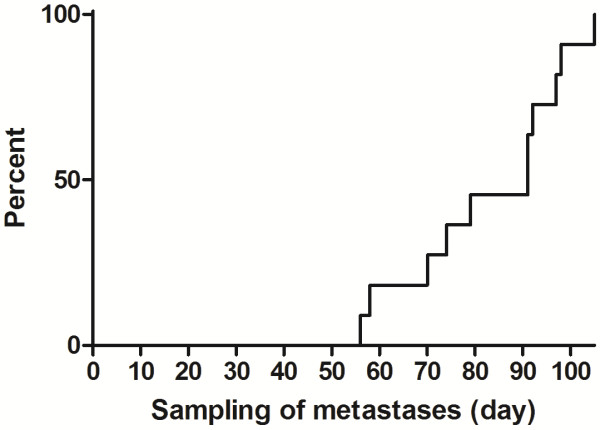
**Timing of the detection of metastatic disease.** Accumulated percentage of rats with metastatic disease observed during the study period (n=11 of 30). At the end of the study, two additional animals had metastases at autopsy; these are not included in this figure. Day 0=day of injection of ^177^Lu-DOTA-BR96.

**Figure 2:**
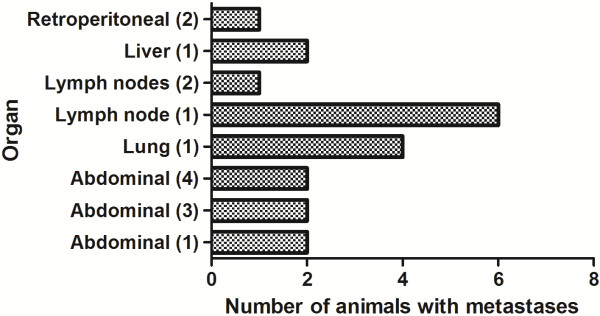
**Tissue localization of detected metastases.** The number of animals with metastases at different locations after treatment with ^177^Lu-DOTA-BR96. Abdominal denotes metastases growing inside the abdominal cavity but not within a specific organ, mostly in the mesentery, in fat tissue or in the peritoneum. Retroperitoneal metastases were found adjacent to the kidneys. The numbers within parentheses are the number of metastases found at the specified location in the same animal.

Of the 35 male BN rats treated with 400 MBq/kg ^177^Lu-DOTA-BR96 (Group A) 30 exhibited consistent CR of the primary tumor. Eleven of these 30 rats developed metastatic disease and metastases were detected in another 2 rats at autopsy at the end of the study. The time at which metastatic disease was observed, and the location of metastases in various organs are presented in Figures 1 and 2. In the untreated group (Group B), all 11 animals were dissected on day 0. None of these animals had visible metastases at autopsy.

### Antigen expression and distribution in tumors

Paraffin embedded tumors and metastases were sectioned and stained with hematoxylin and eosin, and by immunohistochemical staining for the detection of the BR96 target antigen and Ki67, a marker for proliferating cells. In short, 4 μm thick sections were rehydrated and antigen retrieval was performed using the PT Link pre-treatment module (Dako) with acidic target retrieval solution (pH 6), preheated to 65°C and then heated to 99°C for 20 min, followed by rinsing of the slides. The sections were incubated with BR96 or Rabbit anti Ki67 (Clone SP6, NeoMarkers, Fremont, CA) diluted in Antibody diluent (Dako) overnight (BR96) or for 2 h (Ki67) at room temperature. After washing, sections were incubated with anti-human IgG H+L Donkey F(ab)_2_ HRP (Jackson ImmunoResearch Laboratories Inc., West Grove, PA) (for BR96 detection) or Donkey anti-rabbit HRP (Jackson Laboratories) (for Ki67 detection) in Antibody diluent for 3 h (for BR96) or 1 h (for Ki67) at room temperature. Finally, diaminobenzidine (Dako) was added before dehydration and mounting.

Hematoxylin and eosin staining were used to evaluate the overall tumor structure and to distinguish the tumor cells from stromal cells in the tumor. Tumor expression of the BR96 target antigen was scored. A score of 0 corresponds to less than 10% positive (strong complete membranous staining) tumor cells, 1 to 10-50% positive tumor cells, 2 to 50-90% positive tumor cells and 3 to more than 90% positive tumor cells. Proliferation was evaluated in relation to the expression of the BR96 target antigen.

Immunohistochemical staining of the BR96 target antigen was scored from 0 to 3 representing the degree of expression (see Table 1). The results presented for Group A are based on the 13 animals showing consistent CR after treatment of the primary tumor with ^177^Lu-DOTA-BR96 which later developed distant metastases. All the untreated primary tumors in Group B (n=11) had a score of 3, in contrast to the metastases in Group A, in which only 6 out of 23 had a score of 3 (Table 1). The other metastases in Group A had scores of 2 (n=6), 1 (n=9) or 0 (n=2). The metastases with reduced antigen expression showed less BR96 binding, either in specific areas or over the whole tissue section. None of the metastases completely lacked expression of the BR96 target antigen.

**Table 1:** Scores reflecting the BR96 target antigen expression in metastases and primary tumors

**Group**	**Treatment**	**Number of tissue samples analyzed**		**Score 0 < 10 %**^**a**^	**Score 1 10-50 %**^**a**^	**Score 2 50-90 %**^**a**^	**Score 3 > 90 %**^**a**^
A	^177^Lu-DOTA-BR96, with consistent CR	Metastases (from 13 rats)	23	2	9	6	6
B	No treatment	Primary tumors (from 11 rats)	11	0	0	0	11

^a^ % of viable tumor cells showing strong complete membranous staining.

Reduced proliferation was detected in 13 of 23 metastases from rats treated with radioimmunotherapy, but did not show any relation to the expression of the BR96 target antigen. All primary tumors (Group B) showed high proliferation index.

**Figure 3:**
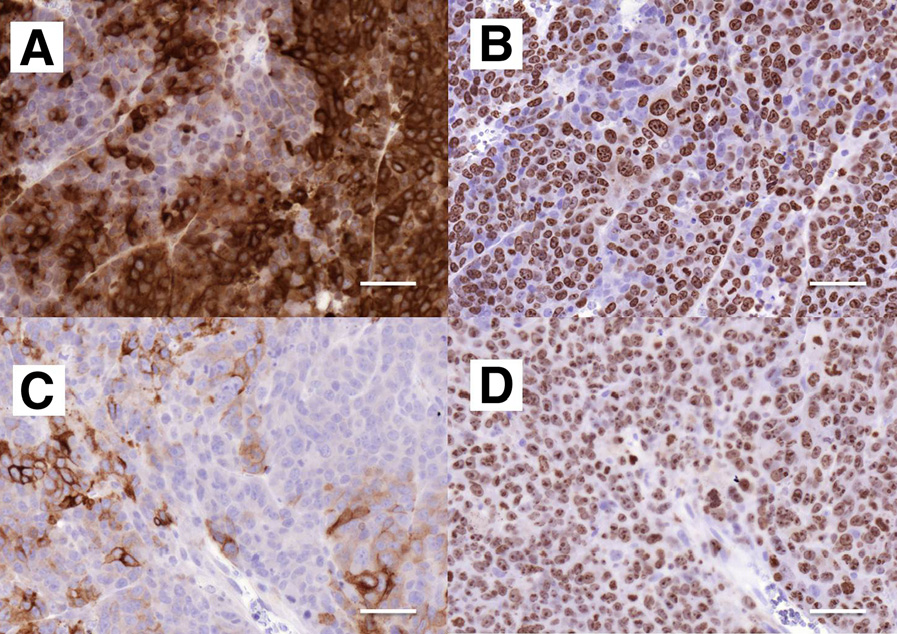
**Different staining pattern in metastases from the same animal.** Stained sections from two different metastases from the same rat treated with ^177^Lu-DOTA-BR96. The metastases were located retroperitoneally adjacent to the right (a and b) and left kidneys (c and d). Staining with BR96 was scored as 2 (a) and as 1 (c), and was not related to proliferation. The results for Ki67 are shown in b and d. Scale bars represent 50 μm.

In half of the cases with more than one stained metastasis sample from the same animal, the BR96 target antigen expression score was the same. An example of different antigen expression within the same animal is illustrated in Figure 3, showing two different metastases adjacent to the right and the left kidneys. Although these two tumors had different BR96 target antigen expression (scores 2 and 1) there was no difference in proliferation.

## Discussion

Primary tumors from untreated animals demonstrated an intense homogeneous BR96 target antigen staining pattern, and all were given a score of 3. In comparison, a score of 3 was only given to 6 of the 23 distant metastases in animals belonging to Group A, showing consistent CR of the primary tumor after treatment with ^177^Lu-DOTA-BR96 (Table 1). The metastases with reduced antigen expression showed less BR96 binding, either in specific areas or over the whole tissue section. None of the metastases completely lacked expression of the BR96 target antigen. It would have been interesting to evaluate metastases in animals not administered radioimmunoconjugate, but this would have required the removal of the primary tumors to prolong the survival of these animals to allow development of metastatic disease. In untreated rats, the tumors reach maximal tolerable volume on day 21-28, i.e. prior to earliest detected metastatic disease (Figure 1). Previous attempts to surgically remove tumors inside the abdomen have not been successful. Apart from technical difficulties, surgical removal of the primary tumor might stimulate the growth of distant metastases [6]. Of the same reason we have not been able to determine whether the antigen expression of the primary tumor becomes more heterogeneous with time and local progression (exceeding 25 x 25 mm) for ethical reasons.

The antigen expression was reduced in 17 of 23 of the analyzed metastases, but it was not possible to determine whether this was due to the metastatic process itself or a result of the radioimmunotherapy, or a combination of both. It is not known whether tumor cells disseminate before, or as a result of, the treatment. However, no visible metastases could be detected at time of treatment.

Since none of the metastases completely lacked the BR96 target antigen, it might be beneficial to repeat radioimmunotherapy with BR96 utilizing a radionuclide with a relatively long range, resulting in irradiation from targeted to adjacent untargeted cells by the cross-fire effect. However, such radionuclides are generally regarded as unsuitable for the treatment of single cells or cell clusters. On the other hand, studies on external beam radiotherapy have indicated that it is not necessary to irradiate all the cells in a tumor to obtain efficacy as a result of the bystander effect [7,8].

In experimental studies of treatment with BR96-doxrubicin antibody conjugates in a rat brain tumor model, Muldoon *et al.*[9] reported changes in the antigen staining pattern in residual tumors. The immunoconjugate treatment resulted in outgrowth of tumors with areas of low or no antigen staining, as well as areas with moderate to intense staining, while the untreated tumors showed uniform intense staining for BR96.

Our findings indicate that during the planning of repeated radioimmunotherapy, and/or radioimmunotherapy in combination with other antibody therapies, it is important to consider the risk of reduced antigen expression in metastases, as this may result in reduced targeting of the therapeutics.

## Conclusions

This study showed that the antigen expression was reduced in 17 of the 23 metastases detected after radioimmunotherapy compared with untreated tumors. Although it was not possible to demonstrate that the antigen reduction is triggered by the radioimmunotherapy this result stress the importance of considering the risk of reduced antigen expression in metastases after radioimmunotherapy prior to further targeted therapies.

## Abbreviations

BN: Brown Norway (inbred rat strain); CR: Complete response; DOTA: S-2-(4-isothiocyanatobenzyl)-1,4,7,10-tetraazacyclododecane tetraacetic acid; HPLC: High performance liquid chromatography; HRP: Horseradish peroxidase; ITLC: Instant thin layer chromatography; Le^y^: Lewis Y blood group antigen; SD: Standard deviation.

## Competing interests

The authors have no affiliations, memberships, funding, or financial holdings that might be perceived as affecting the objectivity of this publication.

## Authors’ contributions

EE and SEE participated in the study conception and design, together carried out the animal experiments, the immunohistochemistry, the data interpretation, and drafted the manuscript. TO performed the radiochemistry. JT and RN participated in the study design and helped to draft the manuscript. All authors read and approved the final manuscript.
